# Evolutions of the Management of Colorectal Cancer Liver Metastasis: A Bibliometric Analysis

**DOI:** 10.7150/jca.52842

**Published:** 2021-04-30

**Authors:** Bao Jin, Xiang'an Wu, Gang Xu, Jiali Xing, Yuxin Wang, Huayu Yang, Shunda Du, Yilei Mao

**Affiliations:** Department of Liver Surgery, Peking Union Medical College Hospital (PUMCH), Peking Union Medical College (PUMC) and Chinese Academy of Medical Sciences (CAMS), Beijing, 100730, China.

**Keywords:** colorectal cancer liver metastasis (CRCLM), bibliometric analysis, therapy, management, citation.

## Abstract

**Background:** Tremendous progress has been made in the treatment of colorectal cancer liver metastasis (CRCLM) in recent decades, and thousands of papers have been published. Therefore, we conducted a bibliometric analysis of articles related to CRCLM treatment to explore its evolution.

**Materials and Methods:** The Clarivate Analytics Web of Science (WOS) Core Collection database was searched through June 2020 using terms related to CRCLM treatment. We analyzed the bibliographic information of the literature related to CRCLM treatment and explored the research topics to understand its evolution over time.

**Results:** We identified 3436 records related to CRCLM treatment in the WOS database. The total number of times these documents were cited ranged 0-2352, and the years of publication spanned 1976-2020. The greatest numbers of articles were published in the United States, Japan, and France. Among institutions, Memorial Sloan-Kettering Cancer Center, MD Anderson Cancer Center, and Oslo University Hospital published the most articles. Regarding authors, Jarnagin WR, Adam R, Vauthey JN published the most articles. The research topics of these articles included systemic chemotherapy, molecular targeted therapy, the outcome of liver resection, prognosis prediction, hepatic artery infusion, radiofrequency ablation, and two-stage hepatectomy.

**Conclusion:** Bibliometric analysis of studies related to CRCLM treatment can help doctors and researchers quickly understand the development trend in this field. These data emphasize the current management of patients with CRCLM, and they can potentially guide the direction of future research.

## Introduction

Colorectal cancer (CRC) is one of the most common malignant tumors, and it is typified by high morbidity and mortality rates globally 1, 2. Liver metastasis is the leading cause of death among patients with CRC, and approximately 50% of patients will develop liver metastases 3, 4. In recent decades, clinicians and researchers have made significant progress in understanding CRC liver metastasis (CRCLM). Therefore, the management and treatment of CRCLM have undergone major changes. Treatments for CRCLM include surgery, interventional therapy, adjuvant chemotherapy, neoadjuvant chemoradiotherapy, and multidisciplinary comprehensive treatment 5, 6. There is a large and growing body of evidence concerning the treatment of CRCLM, with new manuscripts published in various peer-reviewed journals daily. With this large amount of evidence, identifying the most influential manuscripts is difficult.

Bibliometric analysis is an increasingly used method that can analyze the basic characteristics of published literature, including, but not limited to, keywords clustering, the citation network of all manuscripts, and the cooperation network of countries, institutions, and authors of all manuscripts in a field. Via bibliometric analysis of manuscripts in a certain field, we can clearly understand the research hotspots and development trends. Bibliometric analysis has been widely used in various fields of medical research, such as in gastroenterology 7, ophthalmology 8, dermatology 9, respiratory medicine 10, cancer 11, 12, and orthopedics 13. To the best of our knowledge, no bibliometric analysis of the treatment of CRCLM has been reported. In this study, we identified all published studies related to the treatment of CRCLM. The analysis of these data helped us determine the most influential literature in this field, which can enable us to better understand the evolution of the management of CRCLM.

## Materials and Methods

### Literature search and screening

We searched the Clarivate Analytics Web of Science (WOS) Core Collection database which is the most frequently used database for conducting bibliometric analysis studies. The detailed search strategy was as follows: [((Colorectal cancer liver metastasis) OR (CRCLM)) OR (((Colorectal cancer) OR (Colon cancer) OR (rectal cancer)) AND ((liver metastasis) OR (hepatic metastasis)))] AND [(management) OR (treatment) OR (treat) OR (therapeutic) OR (therapy) OR (surgery) OR (surgical) OR (resection) OR (hepatectomy) OR (liver resection) OR (liver transplantation) OR (locoregional therapy) OR (transarterial chemoembolization) OR (TACE) OR (transarterial embolism) OR (TAE) OR (hepatic arterial infusion) OR (HAI) OR (ablation) OR (radiofrequency ablation) OR (RFA) OR (microwave ablation) OR (radiotherapy) OR (stereotactic body radiation therapy) OR (SBRT) OR (selective internal radiation therapy) OR (SIRT) OR (transarterial radioembolization) OR (TARE) OR (Yttrium 90) OR (Y-90) OR (systemic therapy) OR (chemotherapy) OR (adjuvant therapy) OR (targeted therapy) OR (molecular therapy) OR (immunotherapy) OR (cancer vaccines) OR (cell therapy)].

The inclusion criteria of the literature screening were as follows: (1) the main topic of the manuscript was the treatment of CRCLM and the full text was available; (2) the document type was limited to *Article* and the language was limited to English; and (3) the manuscript was published between January 1970 and June 2020. The exclusion criteria were as follows: (1) the main topic was not related to the treatment of CRCLM or it could not be evaluated; and (2) the article was a review, commentary, or case report. Two reviewers (BJ and XA W) independently identified the relevant literature that met the inclusion and exclusion criteria, and any disagreement between the two reviewers was resolved by consensus involving a third reviewer (SD D).

### Data analyses and visualization

After identifying the relevant manuscripts, we downloaded the records including all available information from the WOS Core Collection database. The bibliographic information of the selected publications was converted and analyzed automatically using the bibliometric package (version 3.0.0) in R environment (version 3.6.1), as reported previously 14, 15. The following information was extracted and analyzed using the bibliometric package: title, author, institution, country or region, total number of citations, year of publication, journal, and impact factor. We also determined the main topic, subtopic, and article type of the 100 most cited articles by reading the title, abstract, and full text, if necessary. All raw data will be provided upon reasonable request.

All the information and data for each article were inserted into a spreadsheet and manipulated using Microsoft Excel 2019 (Microsoft Corp., Redmond, WA, USA). Bibexcel was used to calculate the author's h-index, ArcGIS 10.3 was used to produce a global map of countries' publication volumes, and the VOS viewer (Version 1.6.10) was used to produce the institutions' cooperation network map, authors' cooperation network map, keyword clustering map, and density map. An online platform of bibliometric analysis (https://bibliometric.com/) was used to create a cooperation network map among countries. Two researchers independently verified the data entry and analysis.

## Results

### Publication period and citation count

**Figure 1:**
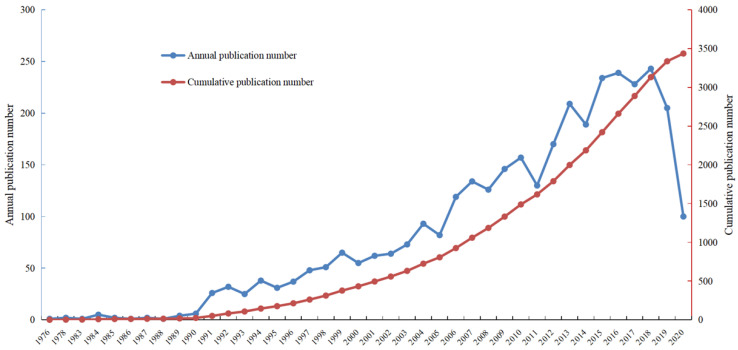
Literature growth curve.

**Figure 2:**
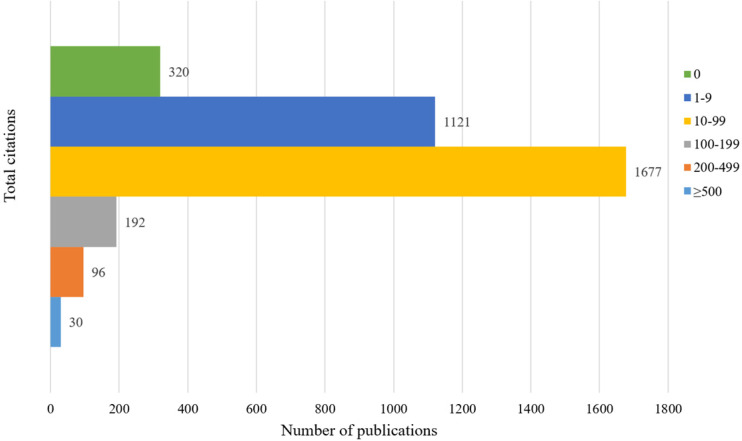
The number of publications by different total citations.

We retrieved a total of 12,947 articles following our search strategy from the Web of Science Core Collection database from January 1970 to June 2020. Based on the inclusion and exclusion criteria of the literature screening, we identified 3436 records focusing on the management of CRCLM. These manuscripts were published between 1976 and 2020, and the total number citations ranged from 0 to 2352. Figure 1 presents the annual number of publications and cumulative number of publications. Between 1970 and 2000, the literature grew slowly, whereas the number of published articles increased dramatically over the last 20 years. The first article on CRCLM treatment was published in *Archives of Surgery* in 1976 (since renamed *JAMA Surgery*) 16. The first author of this article was Dr. Wilson from the University of Minnesota. The researchers summarized the experiences of surgical resection for CRCLM. As of June 2020, this article has been cited 269 times in the WOS Core Collection database. Figure 2 presents the number of citations for the studies. Among 3436 manuscripts, 1121 (32.63%) manuscripts have been cited 1-9 times, 1677 (48.81%) manuscripts have been cited 10-99 times, and 30 (0.87%) manuscripts have been cited more than 500 times. In total, 320 (9.31%) manuscripts have never been cited by other studies, of which 87 were published in 2020. The most cited article, with 2353 citations, was published in the *New England Journal of Medicine* in 2000 17. This multicenter clinical trial introduced adjuvant chemotherapy for metastatic CRC.

### Countries, institutions, and authors

We analyzed the countries or regions, institutions, and authors from which all manuscripts originated to identify the high-impact countries or regions, institutions, and authors in this field.

**Figure 3:**
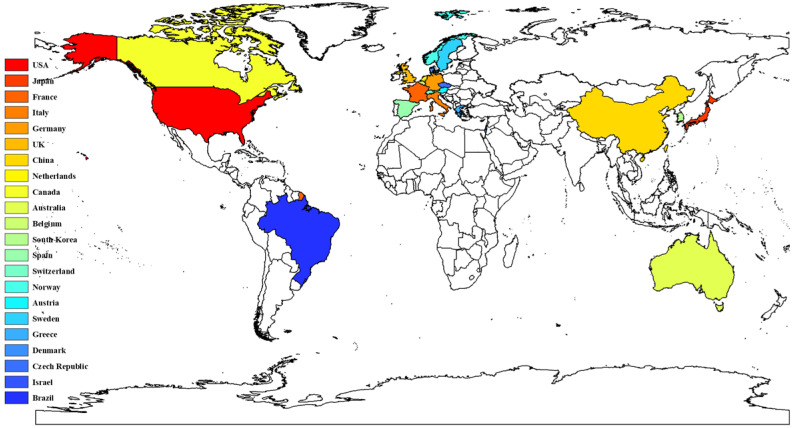
Countries that have published more than 20 articles.

**Figure 4:**
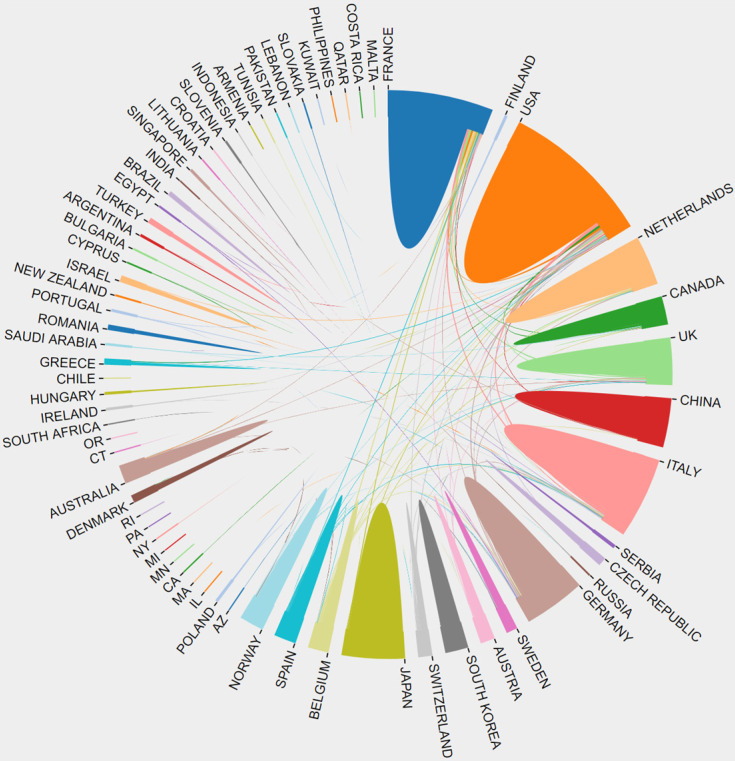
The cooperation relationships among all countries.

In total, 3436 manuscripts on CRCLM treatment were published by researchers from 65 countries or regions. The top 10 high-yield countries with the number of published articles are listed in Table 1. The United States has contributed the most articles and total citations in the field of CRCLM treatment, publishing 821 articles and 57,014 citations. The top 10 high-yield countries included five European countries, two North American countries, two Asian countries, and Australia. France published the third most articles, but the average number of citations was highest for this nation. Australia ranked tenth in the number of articles, but it had the second highest average number of citations. Thus, the quality of research in France and Australia is generally high. The average number of citations for the two Asian countries, namely Japan and China, was much lower than those for other countries. The number of publications, total number of citations, and average number of citations of all countries are presented in Table S1. Figure 3 presents the countries with more than 20 published articles. Cooperation between countries is an important factor in promoting technological development. Figure 4 presents the cooperative relationships among all countries in this field. We can intuitively observe that the United States, France, Netherlands, Germany, the UK, and Italy have close cooperation with other countries.

The top 10 high-yield research institutions are listed in Table 2. Memorial Sloan-Kettering Cancer Center and University of Texas MD Anderson Cancer Center had the largest numbers of published articles and highest number of citations. These institutions published 385 and 169 articles, respectively, and their articles were cited 9211 and 3084 times, respectively. Medical University of Vienna has the highest average number of citations, followed by Paul Brousse Hospital, Memorial Sloan-Kettering Cancer Center, and Institute Gustave Roussy. The average number of citations per article exceeded 20 for these four institutions. Figure S1 presents the results of an analysis of the cooperation network of institutions that have co-published 15 or more articles. In this figure, each dot represents an institution, the size of the dot indicates the number of published articles, and the connection indicates is a cooperative relationship between the institutions. These research institutions were divided into five clusters according to the closeness of cooperation. The detailed information of the institutional partnerships illustrated that Aintree University Hospital NHS Foundation Trust, Medical University of Vienna, Memorial Sloan-Kettering Cancer Center have the most links with other institutions, whereas Hospital Paul-Brousse, Inserter, Aintree University Hospital NHS Foundation Trust, University Medical Center Utrecht, and University of Paris had the highest total link strength (Table S2). In addition, we analyzed the changes of the cooperative relationships between institutions over time. Figure S2 reveals the network of partnerships between institutions before Figure S2A) and after 2010 (Figure S2B). Cooperation between institutions is becoming increasing common, which greatly promotes progress in this field.

In addition, we identified the top 20 most influential authors according to the h-index (Table 3). These 20 authors include 14, 5, and 1 author from the USA, France, and Italy, respectively. Vauthey JN had the highest h-index of 40. He has published 78 articles related to CRCLM treatment, which have been cited 8696 times. Figure S3 presents the analysis of the cooperation network of authors that have co-published 15 or more articles. These authors were divided into more than 10 clusters, among which we identified several major scientific research teams mainly including Vauthey JN, Pawlik TM, Adam R, and Jarnagin WR. The detailed information of the authors' partnerships indicated that Adam R has the most links and Jarnagin WR has the highest total link strength (Table S3).

### Journals

These 3436 manuscripts about the treatment of CRCLM were published in 425 journals. We listed the 10 journals with the most published articles in Table 4. Over the past few decades, *Annals of Surgical Oncology* has published the most manuscripts on CRCLM treatment, including 237 published articles and 3053 citations. Among the 10 journals with the most published articles, five journals had an average number of citations exceeding 10. *Annals of Surgery* had the highest average number of citations per article, exceeding 60. The other four journals were *British Journal of Surgery*, *World Journal of Surgery*, *Annals of Surgical Oncology*, and *Journal of Intestinal Surgery*.

### Analysis of keywords

**Figure 5:**
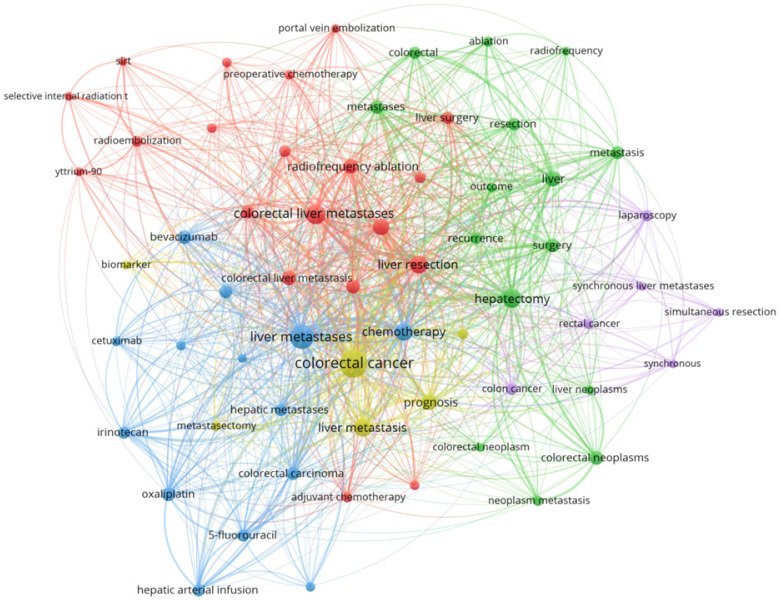
Cluster analysis of keywords that appeared more than 20 times.

To explore the research hotspots in the field of CRCLM treatment, we selected 61 keywords that appeared more than 20 times for cluster analysis (Figure 5). We found that CRCLM treatment-related studies could be roughly divided into the following four categories: surgical treatment, systemic therapy, locoregional therapy, and prognosis. Surgical treatment-related keywords were most common, followed by systemic therapy-related, prognosis-related and locoregional therapy-related keywords, accounting for 35, 27, 20, and 18% of keywords, respectively (Figure S4). The density map of keywords also illustrated that liver resection, chemotherapy, interventional therapy, and prognosis are major hot topics in the field of CRCLM (Figure S5).

### Analysis of the top 100 most cited articles

**Figure 6:**
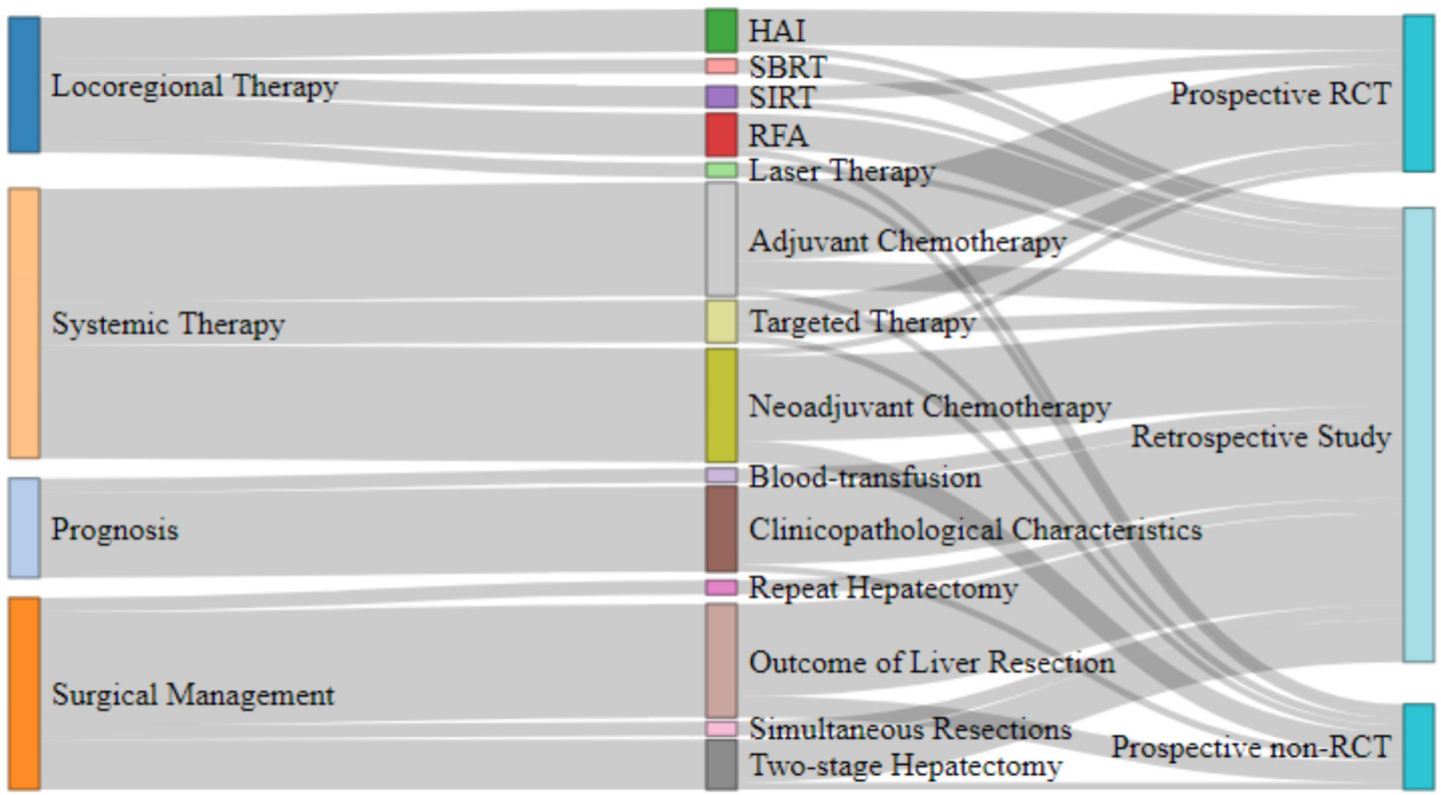
The main topics, subtopics, and article types of the 100 most cited articles.

**Figure 7:**
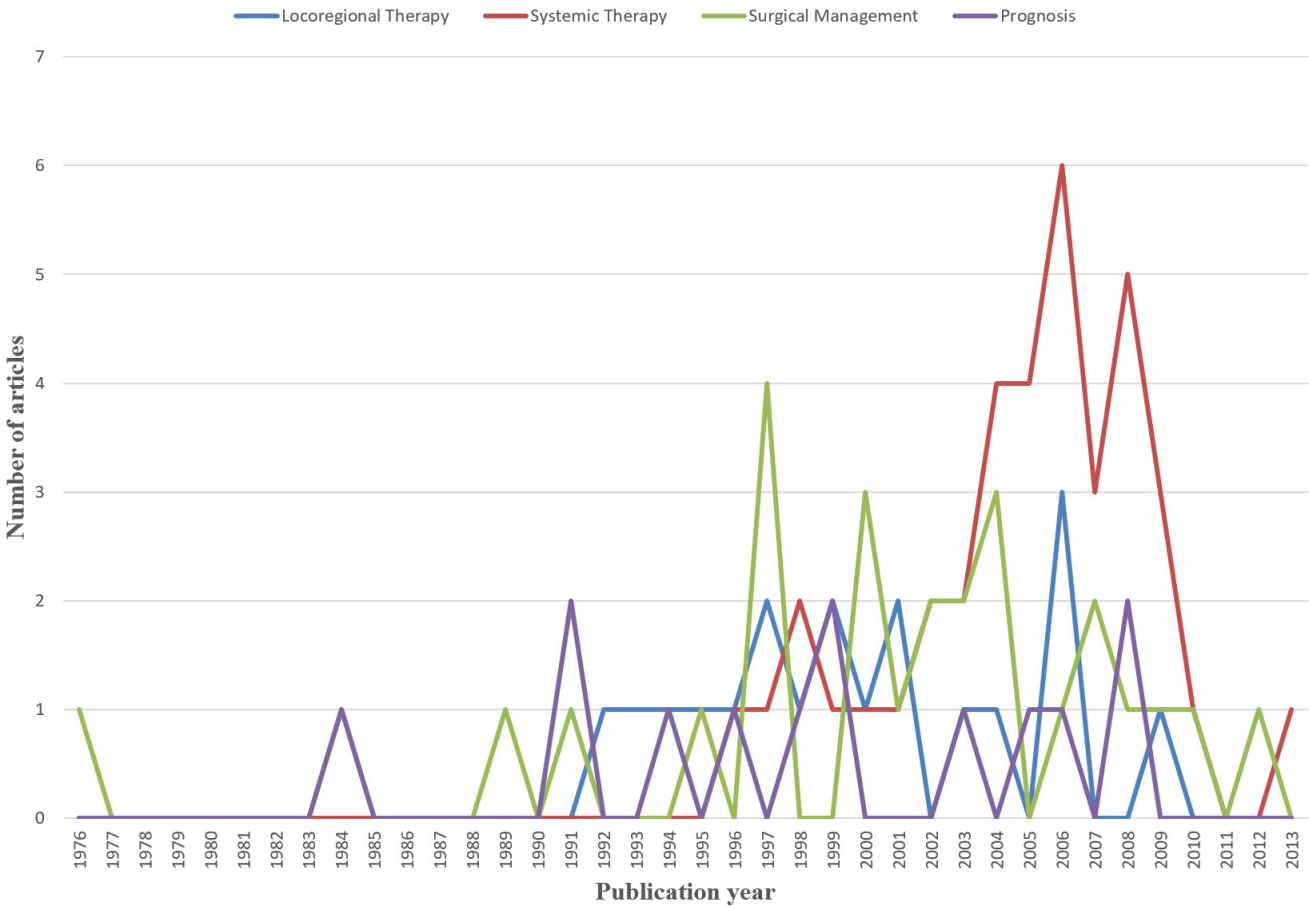
The number of articles of different main topics over the years.

We identified the top 100 most frequently cited articles related to the treatment of CRCLM (Table S4). These 100 articles were published between 1976 and 2016, and the total number of citations ranged from 239 to 2352. *Annals of Surgery* contributed the most frequently cited articles (n = 27), followed by *Journal of Clinical Oncology* (n = 22) and *Annals of Oncology* (n = 8). We analyzed the main topic, subtopic, and article type of the 100 most frequently cited articles related to CRCLM treatment and established the relationships among them (Figure 6). Systemic therapy was the most studied main topic (n = 38), followed by surgical management (n = 27), locoregional therapy (n = 19) and prognosis (n = 14). In addition, two guidelines on the management of CRCLM were published. The outcomes of liver resection, neoadjuvant chemotherapy, and adjuvant chemotherapy were the most studied subtopics (n = 16 each), followed by clinicopathological characteristics (n = 12) and two-stage hepatectomy (n = 7). In terms of article types, the most common was retrospective studies (n = 64), followed by prospective randomized controlled trials (RCTs, n = 22), prospective non-RCTs (n = 12), and guidelines (n = 2). Figure 7 presents the number of articles of different main topics over time. In recent years, the main research topics of the most frequently cited articles have focused on systemic therapy and surgical management, followed by locoregional therapy and prognosis. Articles focusing on systemic therapy have increased over time, which suggests that systemic therapy may become a research hotspot in the next few years.

## Discussion

Globally, CRC is the third most common cancer and fourth leading cause of cancer-related death 1, 2. The most common site of CRC metastasis is the liver, and approximately 50% of patients with CRC will eventually develop liver metastasis, including simultaneous and metachronous metastases 3, 4. Liver metastasis is the main cause of death among patients with CRC. Surgical resection is the main standard treatment for CRCLM, but the resectable rate of CRCLM is less than 20% at presentation 3, 18. In addition to surgical treatment, systemic therapy and locoregional therapy are also important treatments for CRCLM. Because of the rapid development of research on CRCLM management, it may be challenging for researchers and clinicians to identify the most important and influential articles. Bibliometric analysis analyzes the citation history of published papers. It is an important tool to help quantify and evaluate the productivity of the scientific field, and it has been widely used. We created a compendium of articles related to the treatment of CRCLM in the past five decades. We analyzed the basic characteristics of these articles, such as the countries, institutions, authors, journals, and topics of the articles. In particular, we ranked these articles by the total number of citations according to the WOS Core Collection database and analyzed the top 100 most cited articles in this field. Through the analysis of the most frequently cited articles, we discovered the research hotspots of CRCLM treatment in recent decades. In addition, we can identify potential future research directions.

According to the search strategy, we retrieved a total of 12,947 articles. After strict screening according to the inclusion and exclusion criteria, a total of 3,436 articles were identified as studies focusing on the treatment of CRCLM. Shi et al. conducted a bibliometric analysis of articles in the CRCLM field published from 2000 to 2019 based on the WOS database, and identified 7,965 articles 19. Shi et al. were focused on all articles about CRCLM, including basic research, diagnosis, treatment, and prognosis. However, this study only focused on articles about the management of CRCLM to explore the evolution of treatment methods. Therefore, we believe that the number of 3,436 articles we have determined is reasonable.

The analysis of the publication period and number of citations enables us to understand the development of a certain discipline. From the 1970s to the early 2000s, the number of studies on the treatment of CRCLM grew slowly. Over the last 20 years, the amount of research in this field has experienced explosive growth. The number of annual publications has maintained a growing trend. Since our search covered the period to June 2020, the number of studies in 2020 was approximately half of that in 2019. Among the 3436 articles, 3118 (90.75%) articles were cited no more than 100 times, and 318 (9.25%) articles were cited more than 100 times. Studies have found that the year of publication may be related to the number of citations, and the probability of recently published articles being cited is low 12. We identified the contributions of different countries, institutions, and authors to this field. The United States, Japan, France, Italy, Germany, the United Kingdom, and China occupy leading positions in research in this field. Compared with other countries, Japan and China have less cooperation with other countries. The lack of cooperation and communication may be one of the reasons for the low average number of citations for studies published in these two countries. Jarnagin WR, Adam R, Vauthey JN, Gonen M, DeMatteo RP, and Pawlik TM have published the most papers in this field. They have made outstanding contributions to the treatment of CRCLM, which deserves to be identified and remembered. Future research should pay more attention to international multicenter cooperation, and additional prospective multicenter RCTs should be conducted to provide medical evidence for the treatment of CRCLM. Various academic journals have played a vital role in promoting the spread of knowledge. In terms of influence, *Annals of Surgery*, *British Journal of Surgery*, and *Annals of Surgical Oncology* have made the greatest contributions to this field.

The research topics of all articles can be divided into four categories: systemic therapy, locoregional therapy, surgical management, and prognosis. In recent years, the role of systemic therapy in the management of CRCLM has considerably increased. An analysis of the keywords in 3436 records revealed that 27% of keywords were related to systemic therapy. Among the 100 most cited articles, systemic therapy was the most concerned topic (n = 38). For patients with unresectable CRCLM, neoadjuvant chemotherapy can shrink the tumor, possibly permitting surgical resection. Standard chemotherapy with fluorouracil, leucovorin, plus irinotecan or oxaliplatin can promote the conversion of 7%-40% of initially unresectable CRCLM lesions into resectable lesions 20. For patients with resectable CRCLM, adjuvant chemotherapy during the perioperative period can confer survival benefits to patients 21. Molecular targeted therapy combined with chemotherapy provides hope for the treatment of unresectable CRCLM. For patients with initially unresectable RAS-mutant CRCLM, bevacizumab plus modified fluorouracil, leucovorin, and oxaliplatin (mFOLFOX6) can increase the resectability of liver metastases and improve response rates and survival compared with the effects of mFOLFOX6 alone 22. More and more novel therapies for CRCLM are gradually emerging. Immunotherapies such as cancer vaccines and adoptive cell transfer (ACT) therapies have begun to emerge in the field of CRCLM treatment 23. There are many types of cancer vaccines, including DNA vaccines, RNA vaccines, peptide vaccines, dendritic cells (DC) vaccines, etc. 24. Rodriguez et al. conducted a phase II clinical trial of DC vaccine in colon cancer liver metastasis patients with disease-free resection margins. Compared with the control group, the DC group has a clear tendency to reduce and delay tumor recurrence (median disease-free survival, 9.53 months versus 25.26 months) 25. Chimeric antigen receptor T-cell (CAR-T) therapy may be an effective strategy for the treatment of CRCLM. A phase I trial of CAR-T therapy targeting carcinoembryonic antigen (CEA) for patients with metastatic CRC has shown promising results 26. In addition, the therapeutic potential of microRNAs (miRNAs) in CRCLM has also gradually attracted attention. Previous studies have shown that miRNAs can be used as biomarkers for the prognosis of CRCLM patients 27, 28. Future research on miRNAs may help to develop new therapeutic strategies.

Locoregional therapy has also made considerable progress in recent years. Local treatments for CRCLM include hepatic artery infusion chemotherapy (HAIC), radiofrequency ablation (RFA), stereotactic body radiation therapy (SBRT), and selective internal radiation therapy (SIRT). The combination of HAIC and systemic chemotherapy can increase the response rate of patients receiving first-line chemotherapy to more than 90% and increase the response rate of previously treated patients with unresectable CRCLM to 85% 3, 29. Studies have reported that 52% of patients with initially unresectable CRCLM can undergo resection after receiving HAIC combined with systemic chemotherapy 30. For unresectable CRCLM, the long-term results of the recent EORTC-CLOCC trial revealed that RFA (±surgical resection) plus chemotherapy produced an 8-year survival rate of 35.9%, compared with 8.9% for chemotherapy alone 31. SBRT has been used as an effective and safe local therapy in patients with unresectable CRCLM, and it can provide a high local control rate 32, 33. SIRT is a promising novel treatment option 34. The SIRFLOX trial compared the efficacy of SIRT plus systemic chemotherapy and systemic chemotherapy alone in the treatment of unresectable CRCLM. The results revealed that SIRT can prolong progression-free survival and improve response rates in the liver 35. These results indisputably demonstrate that active local treatment can greatly prolong overall survival and even cure some patients.

Research on surgical treatment and prognosis has always been a hotspot in CRCLM. Surgical resection is considered the standard curative option for CRCLM, with an overall 5-year survival rate of 35%-55% after successful resection 36. The expansion of indications for liver resection is based on the improvement of the efficacy of systemic chemotherapy, the improvement of liver surgery techniques, and the expansion of knowledge about liver regeneration 37. The LIGRO Trial found that compared with traditional two-stage hepatectomy, associating liver partition and portal vein ligation for staged hepatectomy can improve the resection rate (92% vs. 57%) without changing the surgical margins, complications rate, and short-term mortality rate 38. Repeated liver resection of CRCLM can achieve comparable perioperative mortality and long-term survival rates as primary liver resection 39. For patients with CRC and synchronous liver metastases, simultaneous resection is safe and feasible 40, 41. Prognosis prediction can help identify patients who are most likely to benefit from various treatments. For many years, a clinical score established by Fong et al. has been widely used to predict the prognosis of patients with CRCLM 42. They identified seven important and independent predictors of poor long-term outcomes: positive margins, extrahepatic disease, node-positive primary disease, disease-free interval from primary to metastases of less than 12 months, more than one hepatic tumor, largest hepatic tumor > 5 cm, and carcinoembryonic antigen level > 200 ng/ml. They used the last five of these criteria to establish a preoperative scoring system that accurately predicts prognosis. Prognosis prediction based on clinicopathological characteristics is increasingly common, and prognosis prediction based on other technologies such as radiomics, genomics, and proteomics is emerging 43-49.

However, it should be noted that there are still some limitations in this study. First, we conducted this bibliometric analysis only based on the WOS core database, without mentioning other databases such as PubMed and PMC. Second, selection bias may appear in literature screening.

In conclusion, in a bibliometric analysis of the literature on CRCLM in the past 50 years, we identified 3436 studies related to the treatment of CRCLM to provide useful insights. We have identified the countries, institutions, authors, and journals that have made significant contributions in this field. We focused on the research topics of these articles to explore the evolution of CRCLM treatment. A single treatment method is about to enter a bottleneck period, and combined treatment with systemic therapy, local therapy, and surgery will become the next research focus to identify possible beneficial treatment combinations. Multidisciplinary approaches including oncologists, surgeons, pathologists, and radiologists should become standard in the treatment of CRCLM. The development of new technologies and new therapies may lead the treatment of CRCLM into a new era.

## Supplementary Material

Supplementary figures and tables.

## Figures and Tables

**Table 1:** Top 10 high-yield countries.

Countries (Regions)	Number of articles	Total citations	Average citations
USA	821	57014	69.44
Japan	482	11098	23.02
France	366	29572	80.80
Italy	359	18934	52.74
Germany	314	18365	58.49
UK	309	16769	54.27
China	256	3921	15.32
Netherlands	186	9488	51.01
Canada	119	6573	55.24
Australia	113	8808	77.95

**Table 2:** Top 10 high-yield institutions.

Institutions	Number of articles	Total citations	Average citations
Memorial Sloan-Kettering Cancer Center	385	9211	23.92
University of Texas MD Anderson Cancer Center	169	3084	18.25
Oslo University Hospital	127	624	4.91
Medical University of Vienna	104	3032	29.15
Institute Gustave Roussy	102	2367	23.21
University of Tokyo	87	1302	14.97
Paul Brousse Hospital	87	2413	27.74
Sun Yat-sen University	86	239	2.78
Fudan University	80	488	6.1
University Medical Center Utrecht	72	670	9.31

**Table 3:** Top 20 most influential authors according to the h-index.

Author	h-index	Country	Total citations	Number of articles	Average citations
Vauthey JN	40	USA	8696	78	111.49
Jarnagin WR	37	USA	5197	81	64.16
Gonen M	36	USA	4921	69	71.32
DeMatteo RP	36	USA	4592	65	70.65
Adam R	35	France	9349	79	118.34
Elias D	34	France	4499	58	77.57
Curley SA	33	USA	7366	40	184.15
Fong Y	31	USA	6362	41	155.17
Blumgart LH	31	USA	7819	37	211.32
Fong YM	30	USA	4512	46	98.09
Capussotti L	28	Italy	4418	44	100.41
D'Angelica M	28	USA	3159	34	92.91
Pawlik TM	27	USA	4774	64	74.59
Castaing D	27	France	6668	41	162.63
Abdalla EK	27	USA	6224	32	194.50
D'Angelica MI	25	USA	1783	56	31.84
Azoulay D	25	France	5737	36	159.36
Ducreux M	25	France	3187	35	91.06
Kemeny N	25	USA	3408	32	106.50
Allen PJ	24	USA	1725	45	38.33

**Table 4:** The top 10 journals with the most published articles in this field.

Journals	Number of articles	Total citations	Average citations	IF (JCR 2019)
*ANNALS OF SURGICAL ONCOLOGY*	227	3053	13.45	4.061
*ANNALS OF SURGERY*	129	8297	64.32	10.130
*EJSO*	114	740	6.49	3.959
*JOURNAL OF SURGICAL ONCOLOGY*	113	819	7.25	2.771
*HEPATO-GASTROENTEROLOGY*	109	379	3.48	-
*BRITISH JOURNAL OF SURGERY*	107	2548	23.81	5.676
*JOURNAL OF GASTROINTESTINAL SURGERY*	106	1141	10.76	2.573
*WORLD JOURNAL OF SURGERY*	92	1306	14.2	2.234
*HPB*	89	458	5.15	3.401
*ANTICANCER RESEARCH*	84	297	3.54	1.994

IF, Impact Factor. JCR, Journal Citations Reports.
