# Supplementary material for: Evolutions of the Management of Colorectal Cancer Liver Metastasis: A Bibliometric Analysis
Source: J Cancer. 2021 Apr 30;12(12):3660–70. doi: 10.7150/jca.52842 (PMC8120180; doi:10.7150/jca.52842)
Supplement: Supplementary file 1 — Supplementary figures and tables. [file jcav12p3660s1.pdf]

Figure S1: The cooperation relationships of institutions that have co-published more than fifteen articles.

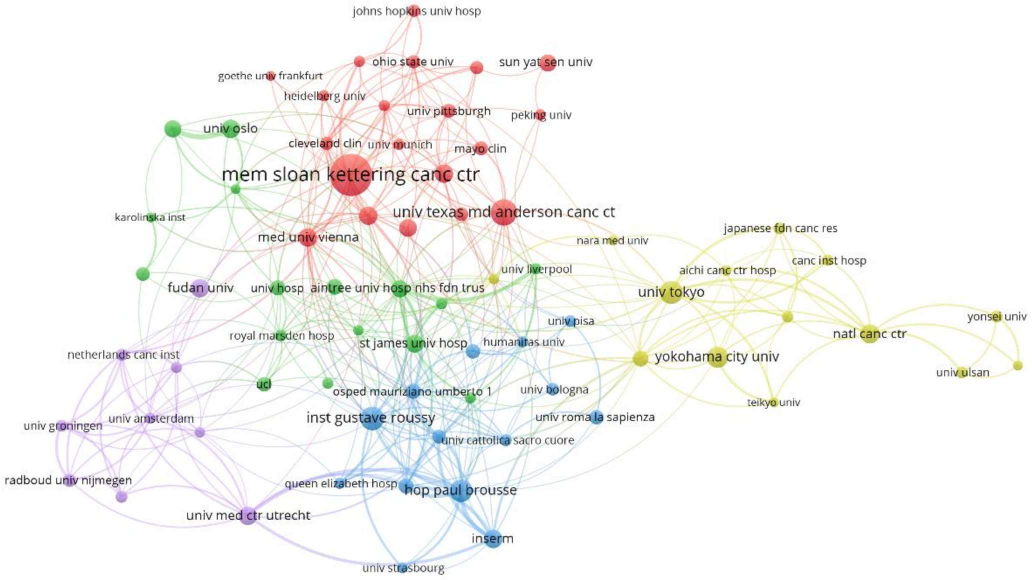

Figure S2: The network of partnerships between institutions before 2010 (Fig S2A) and after 2010 (Fig S2B).

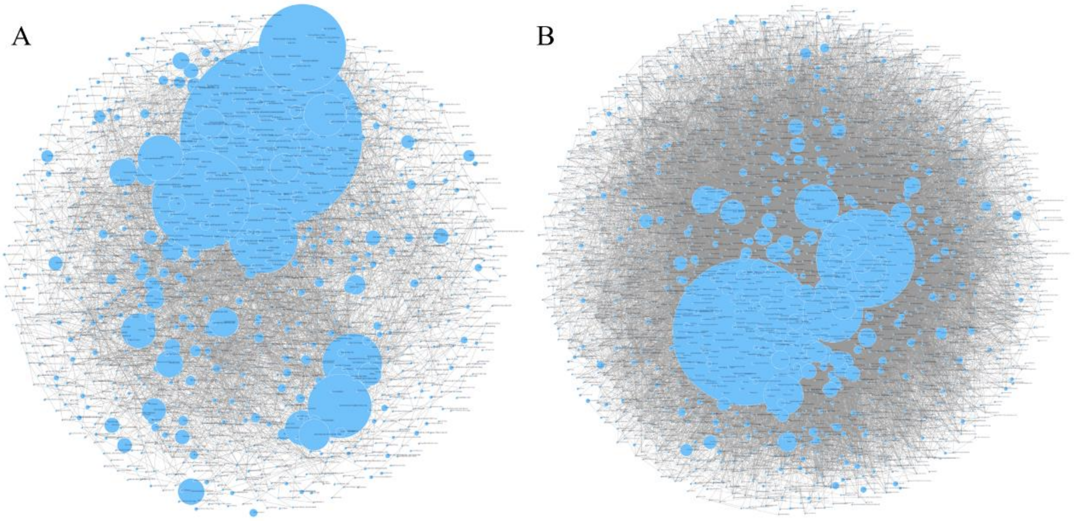

Figure S3: The cooperation relationships of authors that have co-published more than fifteen articles.

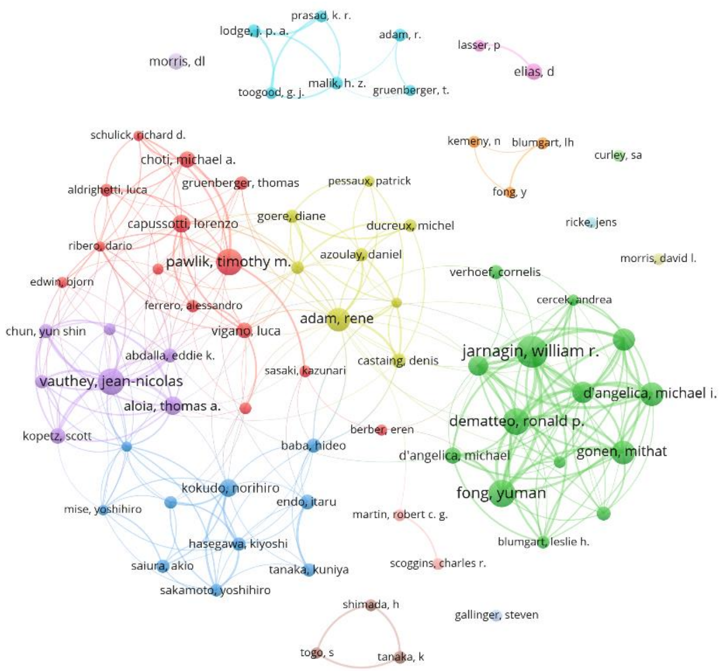

Figure S4: The proportion of the four types of keywords in all manuscripts.

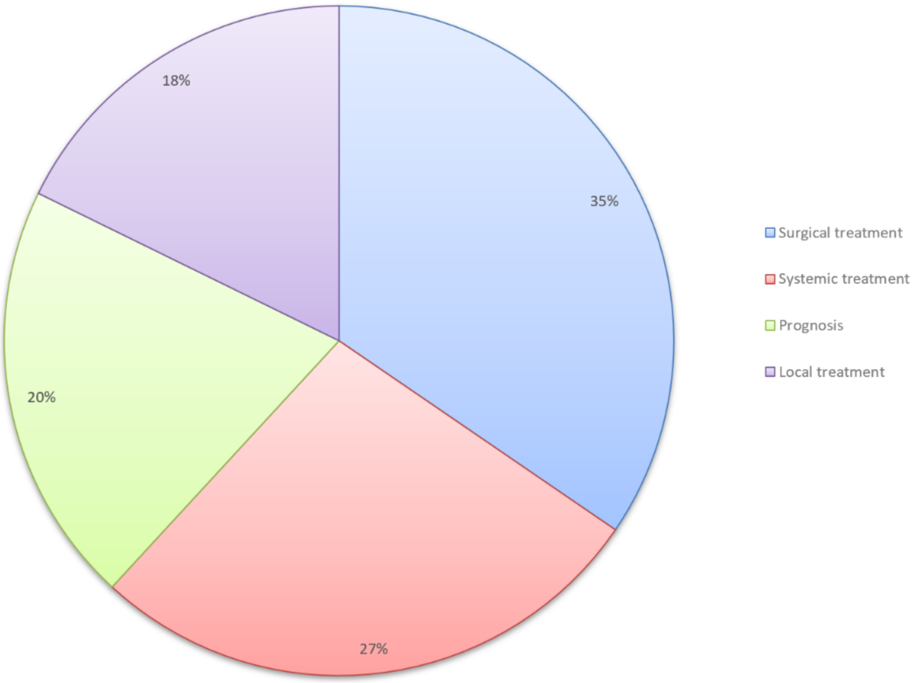

Figure S5: The density map of keywords.

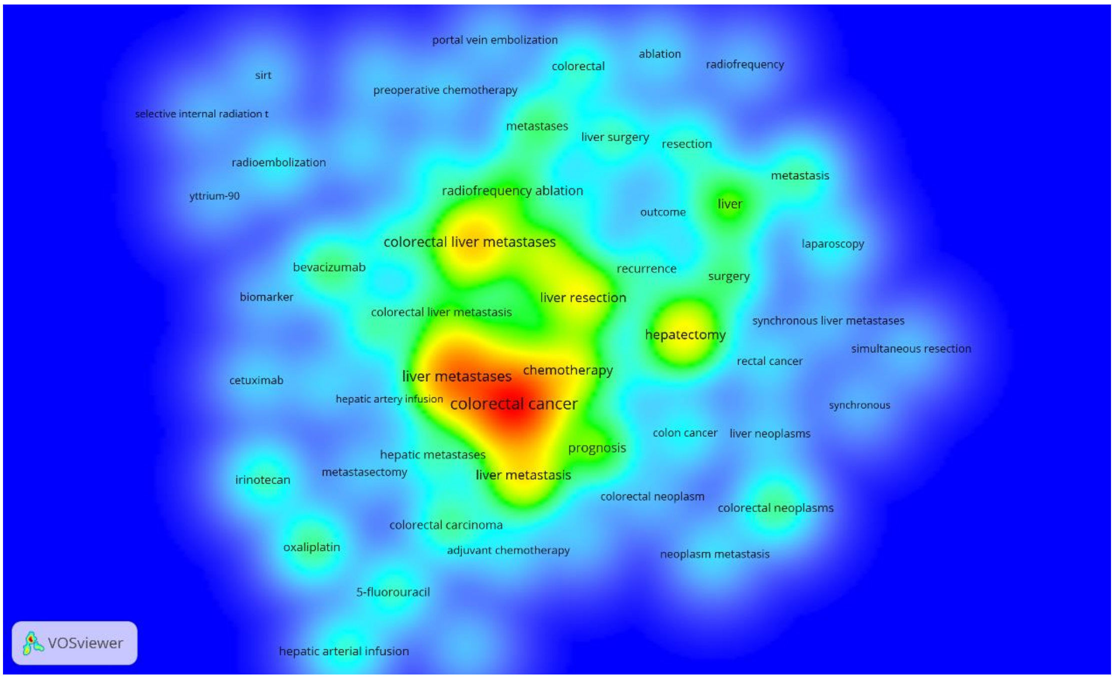

Table S1: The number of publications and the citations of all countries.

Countries (Regions)	Number of articles	Total citations	Average citations
USA	821	57014	69.44
Japan	482	11098	23.02
France	366	29572	80.80
Italy	359	18934	52.74
Germany	314	18365	58.49
UK	309	16769	54.27
China	256	3921	15.32
Netherlands	186	9488	51.01
Canada	119	6573	55.24
Australia	113	8808	77.95
Belgium	105	8941	85.15
South Korea	90	1513	16.81
Spain	84	5060	60.24
Switzerland	74	6598	89.16
Norway	70	4396	62.80
Austria	65	6217	95.65
Sweden	54	4085	75.65
Greece	38	1236	32.53
Denmark	33	1738	52.67
Czech Republic	30	584	19.47
Israel	28	1929	68.89
Brazil	21	974	46.38
Turkey	18	145	8.06
Portugal	16	1402	87.63
Romania	15	142	9.47
Finland	14	2127	151.93
New Zealand	14	520	37.14
POLAND	14	155	11.07
Argentina	12	289	24.08
Serbia	12	375	31.25
Egypt	11	155	14.09
Saudi Arabia	10	92	9.20
Ireland	10	168	16.80
Hungary	9	969	107.67
Russia	9	341	37.89
Singapore	8	90	11.25
Slovenia	7	156	22.29
Croatia	6	63	10.50
Bulgaria	6	19	3.17
Cyprus	4	814	203.50
South Africa	3	166	55.33

India	3	7	2.33
Slovakia	2	8	4.00
UKRAINE	2	98	49.00
Qatar	2	127	63.50
Armenia	2	3	1.50
FED REP GER	2	5	2.50
Costa Rica	2	6	3.00
Philippines	2	239	119.50
Tunisia	2	0	0.00
Thailand	1	17	17.00
Cameroon	1	7	7.00
Chile	1	2	2.00
Estonia	1	39	39.00
IRAQ	1	6	6.00
Mexico	1	1	1.00
Albania	1	1	1.00
Malta	1	30	30.00
Kuwait	1	8	8.00
Lebanon	1	58	58.00
Lithuania	1	3	3.00
U Arab Emirates	1	0	0.00
Iran	1	0	0.00
Pakistan	1	0	0.00
Indonesia	1	0	0.00

Table S2: The detailed information of the institutional partnership.

Institutions	Clusters	Links	Total link strength	Number of articles	Total citations	Average citations
mem sloan kettering canc ctr	1	21	38	167	16067	96.21
univ texas md anderson canc ctr	1	18	25	76	4155	54.67
inst gustave roussy	3	17	47	64	5204	81.31
univ tokyo	4	18	57	60	2064	34.40
hop paul brousse	3	20	99	58	5611	96.74
yokohama city univ	4	9	24	52	1381	26.56
natl canc ctr	4	11	43	43	1693	39.37
med univ vienna	1	23	44	42	3229	76.88
johns hopkins univ	1	18	38	42	2080	49.52
univ med ctr utrecht	5	12	62	42	1744	41.52
inserm	3	11	77	41	2697	65.78
univ louisville	1	9	11	41	1342	32.73
univ oslo	2	5	37	41	949	23.15
fudan univ	5	3	3	41	806	19.66
univ toronto	1	6	8	40	1914	47.85
st james univ hosp	2	7	7	38	1761	46.34
aintree univ hosp nhs fdn trust	2	25	65	37	3243	87.65
oslo univ hosp	2	7	37	36	729	20.25
sun yat sen univ	1	2	2	36	476	13.22
kumamoto univ	4	15	41	32	496	15.50
univ texas	2	6	8	31	6432	207.48
osped mauriziano umberto 1	3	18	45	30	1879	62.63
univ hosp	2	12	16	30	2106	70.20

univ paris sud	3	16	60	29	3799	131.00
ucl	2	8	12	28	1606	57.36
univ milan	3	8	16	27	1033	38.26
univ roma la sapienza	3	3	7	27	1651	61.15
univ paris 11	3	18	58	26	1576	60.62
harvard univ	1	7	7	26	4044	155.54
univ pittsburgh	1	7	8	25	1408	56.32
mayo clin	1	6	10	25	2131	85.24
lund univ	2	5	9	25	494	19.76
ohio state univ	1	13	18	24	438	18.25
cleveland clin	1	12	21	24	896	37.33
radboud univ nijmegen	5	7	28	24	634	26.42
duke univ	1	7	11	24	3282	136.75
univ bologna	3	5	8	21	288	13.71
johns hopkins univ hosp	1	4	7	21	1581	75.29
royal marsden hosp	2	12	17	20	2558	127.90
netherlands canc inst	5	11	35	20	362	18.10
univ cattolica sacro cuore	3	11	22	20	650	32.50
univ pisa	3	7	11	20	1699	84.95
yonsei univ	4	3	13	20	349	17.45
univ ulsan	4	3	12	20	260	13.00
univ groningen	5	7	31	19	544	28.63
leiden univ	5	7	23	19	599	31.53
japanese fdn canc res	4	6	22	19	517	27.21
maastricht univ	5	12	29	18	231	12.83
univ hosp geneva	2	12	18	18	1897	105.39

ctr leon berard	2	9	18	18	385	21.39
canc inst hosp	4	6	17	18	1064	59.11
univ munich	1	3	5	18	720	40.00
univ amsterdam	5	14	34	17	578	34.00
stanford univ	1	12	22	17	834	49.06
univ liverpool	2	11	27	17	308	18.12
univ athens	1	9	16	17	264	15.53
queen elizabeth hosp	3	9	15	17	580	34.12
tokyo med & dent univ	4	8	18	17	419	24.65
humanitas univ	3	6	11	17	243	14.29
heidelberg univ	1	6	7	17	762	44.82
peking univ	1	4	4	17	201	11.82
univ london imperial coll sci technol & med	2	2	4	17	954	56.12
erasmus mc	5	14	18	16	604	37.75
hop ambroise pare	2	14	17	16	3134	195.88
univ paris 05	4	11	16	16	759	47.44
aichi canc ctr hosp	4	9	16	16	216	13.50
univ strasbourg	3	4	15	16	582	36.38
haukeland hosp	2	13	27	15	363	24.20
teikyo univ	4	5	21	15	296	19.73
karolinska inst	2	5	12	15	1580	105.33
nara med univ	4	4	7	15	214	14.27
sungkyunkwan univ	4	3	13	15	284	18.93
goethe univ frankfurt	1	3	3	15	1384	92.27

Table S3: The detailed information of the author’s partnership.

Authors	Clusters	Links	Total link strength
adam, rene	4	25	100
vauthey, jean-nicolas	5	19	159
jarnagin, william r.	2	15	354
kingham, t. peter	2	15	197
capussotti, lorenzo	1	15	74
aloia, thomas a.	5	14	96
fong, yuman	2	13	241
dematteo, ronald p.	2	13	303
gonen, mithat	2	13	214
allen, peter j.	2	13	242
kemeny, nancy e.	2	13	190
kokudo, norihiro	3	13	74
giuliante, felice	1	13	19
shindoh, junichi	3	13	53
d'angelica, michael i.	2	12	251
d'angelica, michael	2	12	94
vigano, luca	1	12	48
shia, jinru	2	12	96
cercek, andrea	2	12	83
ribero, dario	1	12	57
pawlik, timothy m.	1	11	76
abdalla, eddie k.	5	11	69
goere, diane	4	11	48
mise, yoshihiro	3	11	32
kopetz, scott	5	10	73
baba, hideo	3	10	25
sofocleous, constantinos t.	2	10	40
chun, yun shin	5	10	71
blumgart, leslie h.	2	10	98
curley, steven a.	5	9	68
gruenberger, thomas	1	9	12
hasegawa, kiyoshi	3	9	63
elias, dominique	4	9	41
sakamoto, yoshihiro	3	9	56
aldrighetti, luca	1	9	29
makuuchi, masatoshi	3	9	36
conrad, claudius	5	9	50
choti, michael a.	1	8	54
azoulay, daniel	4	8	40
endo, itaru	3	7	30
ferrero, alessandro	1	7	30
torzilli, guido	1	7	15

cunha, antonio sa	4	7	36
castaing, denis	4	6	50
saiura, akio	3	6	26
edwin, bjorn	1	6	7
schulick, richard d.	1	6	37
verhoef, cornelis	2	5	11
tanaka, kuniya	3	5	27
ducreux, michel	4	5	33
malik, h. z.	6	5	34
sasaki, kazunari	1	4	17
pessaux, patrick	4	4	19
lodge, j. p. a.	6	3	44
toogood, g. j.	6	3	43
prasad, k. r.	6	3	38
berber, eren	1	3	3
adam, r.	6	2	6
tanaka, k	8	2	33
kemeny, n	7	2	6
martin, robert c. g.	10	2	11
blumgart, lh	7	2	11
shimada, h	8	2	33
fong, y	7	2	13
gruenberger, t.	6	2	5
togo, s	8	2	32
elias, d	9	1	17
lasser, p	9	1	17
scoggins, charles r.	10	1	10
morris, dl	14	0	0
gallinger, steven	12	0	0
morris, david l.	13	0	0
curley, sa	11	0	0
ricke, jens	15	0	0

Table S4: The top 100 most frequently cited articles related to the treatment of CRCLM.

Rank	Articles	Total citations
1	Saltz LB, et al. Irinotecan plus fluorouracil and leucovorin for metastatic colorectal cancer. Irinotecan Study Group. *N Engl J Med*. 2000;343(13):905-914.	2352
2	Fong Y, et al. Clinical score for predicting recurrence after hepatic resection for metastatic colorectal cancer: analysis of 1001 consecutive cases. *Ann Surg*. 1999;230(3):309-321.	2349
3	Kabbinavar F, Hurwitz HI, Fehrenbacher L, et al. Phase II, randomized trial comparing bevacizumab plus fluorouracil (FU)/leucovorin (LV) with FU/LV alone in patients with metastatic colorectal cancer. *J Clin Oncol*. 2003;21(1):60-65.	1254
4	Nordlinger B, et al. Surgical resection of colorectal carcinoma metastases to the liver. A prognostic scoring system to improve case selection, based on 1568 patients. Association Française de Chirurgie. *Cancer*. 1996;77(7):1254-1262.	1240
5	Nordlinger B, et al. Perioperative chemotherapy with FOLFOX4 and surgery versus surgery alone for resectable liver metastases from colorectal cancer (EORTC Intergroup trial 40983): a randomised controlled trial. *Lancet*. 2008;371(9617):1007-1016.	1229
6	Abdalla EK, et al. Recurrence and outcomes following hepatic resection, radiofrequency ablation, and combined resection/ablation for colorectal liver metastases. *Ann Surg*. 2004;239(6):818-827.	1139
7	Scheele J, et al. Resection of colorectal liver metastases. *World J Surg*. 1995;19(1):59-71.	1133
8	Choti MA, et al. Trends in long-term survival following liver resection for hepatic colorectal metastases. *Ann Surg*. 2002;235(6):759-766.	1016
9	Cunningham D, et al. Randomised trial of irinotecan plus supportive care versus supportive care alone after fluorouracil failure for patients with metastatic colorectal cancer. *Lancet*. 1998;352(9138):1413-1418.	1004
10	Adam R, et al. Rescue surgery for unresectable colorectal liver metastases downstaged by chemotherapy: a model to predict long-term survival. *Ann Surg*. 2004;240(4):644-658.	990
11	Fong Y, et al. Liver resection for colorectal metastases. *J Clin Oncol*. 1997;15(3):938-946.	893

12	Vauthey JN, et al. Chemotherapy regimen predicts steatohepatitis and an increase in 90-day mortality after surgery for hepatic colorectal metastases. *J Clin Oncol*. 2006;24(13):2065-2072.	863
13	Curley SA, et al. Radiofrequency ablation of unresectable primary and metastatic hepatic malignancies: results in 123 patients. *Ann Surg*. 1999;230(1):1-8.	847
14	Rougier P, et al. Randomised trial of irinotecan versus fluorouracil by continuous infusion after fluorouracil failure in patients with metastatic colorectal cancer [published correction appears in Lancet 1998 Nov 14;352(9140):1634]. *Lancet*. 1998;352(9138):1407-1412.	807
15	Van Cutsem E, et al. ESMO consensus guidelines for the management of patients with metastatic colorectal cancer. *Ann Oncol*. 2016;27(8):1386-1422.	801
16	Falcone A, et al. Phase III trial of infusional fluorouracil, leucovorin, oxaliplatin, and irinotecan (FOLFOXIRI) compared with infusional fluorouracil, leucovorin, and irinotecan (FOLFIRI) as first-line treatment for metastatic colorectal cancer: the Gruppo Oncologico Nord Ovest. *J Clin Oncol*. 2007;25(13):1670-1676.	742
17	Pawlik TM, et al. Effect of surgical margin status on survival and site of recurrence after hepatic resection for colorectal metastases. *Ann Surg*. 2005;241(5):715-724.	729
18	Kopetz S, et al. Improved survival in metastatic colorectal cancer is associated with adoption of hepatic resection and improved chemotherapy. *J Clin Oncol*. 2009;27(22):3677-3683.	726
19	Bismuth H, et al. Resection of nonresectable liver metastases from colorectal cancer after neoadjuvant chemotherapy. *Ann Surg*. 1996;224(4):509-522.	719
20	Kabbinavar FF, et al. Addition of bevacizumab to bolus fluorouracil and leucovorin in first-line metastatic colorectal cancer: results of a randomized phase II trial. *J Clin Oncol*. 2005;23(16):3697-3705.	694
21	Tomlinson JS, et al. Actual 10-year survival after resection of colorectal liver metastases defines cure. *J Clin Oncol*. 2007;25(29):4575-4580.	669
22	Rubbia-Brandt L, et al. Severe hepatic sinusoidal obstruction associated with oxaliplatin-based chemotherapy in patients with metastatic colorectal cancer. *Ann Oncol*. 2004;15(3):460-466.	669
23	Solbiati L, et al. Percutaneous radio-frequency ablation of hepatic metastases from colorectal cancer: long-term results in 117 patients. *Radiology*. 2001;221(1):159-166.	662

24	Kemeny N, et al. Hepatic arterial infusion of chemotherapy after resection of hepatic metastases from colorectal cancer. *N Engl J Med*. 1999;341(27):2039-2048.	639
25	Schnitzbauer AA, et al. Right portal vein ligation combined with in situ splitting induces rapid left lateral liver lobe hypertrophy enabling 2-staged extended right hepatic resection in small-for-size settings. *Ann Surg*. 2012;255(3):405-414.	625
26	Rees M, et al. Evaluation of long-term survival after hepatic resection for metastatic colorectal cancer: a multifactorial model of 929 patients. *Ann Surg*. 2008;247(1):125-135.	624
27	Folprecht G, et al. Tumour response and secondary resectability of colorectal liver metastases following neoadjuvant chemotherapy with cetuximab: the CELIM randomised phase 2 trial. *Lancet Oncol*. 2010;11(1):38-47.	600
28	Minagawa M, et al. Extension of the frontiers of surgical indications in the treatment of liver metastases from colorectal cancer: long-term results. *Ann Surg*. 2000;231(4):487-499.	519
29	Adam R, et al. Tumor progression while on chemotherapy: a contraindication to liver resection for multiple colorectal metastases?. *Ann Surg*. 2004;240(6):1052-1064.	513
30	Adam R, et al. Five-year survival following hepatic resection after neoadjuvant therapy for nonresectable colorectal. *Ann Surg Oncol*. 2001;8(4):347-353.	512
31	Karoui M, et al. Influence of preoperative chemotherapy on the risk of major hepatectomy for colorectal liver metastases. *Ann Surg*. 2006;243(1):1-7.	493
32	Lévi F, et al. Randomised multicentre trial of chronotherapy with oxaliplatin, fluorouracil, and folinic acid in metastatic colorectal cancer. International Organization for Cancer Chronotherapy. *Lancet*. 1997;350(9079):681-686.	473
33	Goldberg SN, et al. Treatment of intrahepatic malignancy with radiofrequency ablation: radiologic-pathologic correlation. *Cancer*. 2000;88(11):2452-2463.	471
34	Giacchetti S, et al. Long-term survival of patients with unresectable colorectal cancer liver metastases following infusional chemotherapy with 5-fluorouracil, leucovorin, oxaliplatin and surgery. *Ann Oncol*. 1999;10(6):663-669.	471

35	Rougier P, et al. Hepatic arterial infusion of floxuridine in patients with liver metastases from colorectal carcinoma: long-term results of a prospective randomized trial. *J Clin Oncol*. 1992;10(7):1112-1118.	469
36	Adson MA, et al. Resection of hepatic metastases from colorectal cancer. *Arch Surg*. 1984;119(6):647-651.	468
37	Adam R, et al. Two-stage hepatectomy: A planned strategy to treat irresectable liver tumors. *Ann Surg*. 2000;232(6):777-785.	467
38	Scheele J, et al. Indicators of prognosis after hepatic resection for colorectal secondaries. *Surgery*. 1991;110(1):13-29.	460
39	Van Cutsem E, et al. Metastatic colorectal cancer: ESMO Clinical Practice Guidelines for diagnosis, treatment and follow-up [published correction appears in Ann Oncol. 2015 Sep;26 Suppl 5:v174-7]. *Ann Oncol*. 2014;25 Suppl 3:iii1-iii9.	456
40	Solbiati L, et al. Percutaneous US-guided radio-frequency tissue ablation of liver metastases: treatment and follow-up in 16 patients. *Radiology*. 1997;202(1):195-203.	451
41	Fortner JG, et al. Multivariate analysis of a personal series of 247 consecutive patients with liver metastases from colorectal cancer. I. Treatment by hepatic resection. *Ann Surg*. 1984;199(3):306-316.	451
42	Nordlinger B, et al. Perioperative FOLFOX4 chemotherapy and surgery versus surgery alone for resectable liver metastases from colorectal cancer (EORTC 40983): long-term results of a randomised, controlled, phase 3 trial. *Lancet Oncol*. 2013;14(12):1208-1215.	451
43	Steele G Jr, et al. Resection of hepatic metastases from colorectal cancer. Biologic perspective. *Ann Surg*. 1989;210(2):127-138.	450
44	Folprecht G, et al. Neoadjuvant treatment of unresectable colorectal liver metastases: correlation between tumour response and resection rates. *Ann Oncol*. 2005;16(8):1311-1319.	450
45	Allen-Mersh TG, et al. Quality of life and survival with continuous hepatic-artery floxuridine infusion for colorectal liver metastases. *Lancet*. 1994;344(8932):1255-1260.	429
46	Fernandez FG, et al. Five-year survival after resection of hepatic metastases from colorectal cancer in patients screened by positron emission tomography with F-18 fluorodeoxyglucose (FDG-PET). *Ann Surg*. 2004;240(3):438-450.	428

47	Gayowski TJ, et al. Experience in hepatic resection for metastatic colorectal cancer: analysis of clinical and pathologic risk factors. *Surgery*. 1994;116(4):703-711.	419
48	de Jong MC, et al. Rates and patterns of recurrence following curative intent surgery for colorectal liver metastasis: an international multi-institutional analysis of 1669 patients. *Ann Surg*. 2009;250(3):440-448.	415
49	Kooby DA, et al. Influence of transfusions on perioperative and long-term outcome in patients following hepatic resection for colorectal metastases. *Ann Surg*. 2003;237(6):860-870.	413
50	Benoist S, et al. Complete response of colorectal liver metastases after chemotherapy: does it mean cure?. *J Clin Oncol*. 2006;24(24):3939-3945.	401
51	Azoulay D, et al. Resection of nonresectable liver metastases from colorectal cancer after percutaneous portal vein embolization. *Ann Surg*. 2000;231(4):480-486.	383
52	Blazer DG 3rd, et al. Pathologic response to preoperative chemotherapy: a new outcome end point after resection of hepatic colorectal metastases. *J Clin Oncol*. 2008;26(33):5344-5351.	380
53	Portier G, et al. Multicenter randomized trial of adjuvant fluorouracil and folinic acid compared with surgery alone after resection of colorectal liver metastases: FFCD ACHBTH AURC 9002 trial. *J Clin Oncol*. 2006;24(31):4976-4982.	371
54	Doci R, et al. One hundred patients with hepatic metastases from colorectal cancer treated by resection: analysis of prognostic determinants. *Br J Surg*. 1991;78(7):797-801.	369
55	Gray B, et al. Randomised trial of SIR-Spheres plus chemotherapy vs. chemotherapy alone for treating patients with liver metastases from primary large bowel cancer. *Ann Oncol*. 2001;12(12):1711-1720.	368
56	Livraghi T, et al. Saline-enhanced radio-frequency tissue ablation in the treatment of liver metastases. *Radiology*. 1997;202(1):205-210.	362
57	Aloia T, et al. Liver histology and surgical outcomes after preoperative chemotherapy with fluorouracil plus oxaliplatin in colorectal cancer liver metastases. *J Clin Oncol*. 2006;24(31):4983-4990.	358
58	Meta-Analysis Group in Cancer, et al. Reappraisal of hepatic arterial infusion in the treatment of nonresectable liver metastases from colorectal cancer. *J Natl Cancer Inst*. 1996;88(5):252-258.	357

59	Alberts SR, et al. Oxaliplatin, fluorouracil, and leucovorin for patients with unresectable liver-only metastases from colorectal cancer: a North Central Cancer Treatment Group phase II study. *J Clin Oncol*. 2005;23(36):9243-9249.	350
60	Jaeck D, et al. A two-stage hepatectomy procedure combined with portal vein embolization to achieve curative resection for initially unresectable multiple and bilobar colorectal liver metastases. *Ann Surg*. 2004;240(6):1037-1051.	347
61	Mitry E, et al. Adjuvant chemotherapy after potentially curative resection of metastases from colorectal cancer: a pooled analysis of two randomized trials. *J Clin Oncol*. 2008;26(30):4906-4911.	347
62	Köhne CH, et al. Clinical determinants of survival in patients with 5-fluorouracil-based treatment for metastatic colorectal cancer: results of a multivariate analysis of 3825 patients. *Ann Oncol*. 2002;13(2):308-317.	339
63	Cady B, et al. Surgical margin in hepatic resection for colorectal metastasis: a critical and improvable determinant of outcome. *Ann Surg*. 1998;227(4):566-571.	336
64	Adam R, et al. Patients with initially unresectable colorectal liver metastases: is there a possibility of cure?. *J Clin Oncol*. 2009;27(11):1829-1835.	329
65	Rosen CB, et al. Perioperative blood transfusion and determinants of survival after liver resection for metastatic colorectal carcinoma. *Ann Surg*. 1992;216(4):493-505.	328
66	de Baère T, et al. Adverse events during radiofrequency treatment of 582 hepatic tumors. *AJR Am J Roentgenol*. 2003;181(3):695-700.	323
67	Hemming AW, et al. Preoperative portal vein embolization for extended hepatectomy. *Ann Surg*. 2003;237(5):686-693.	319
68	Gruenberger B, et al. Bevacizumab, capecitabine, and oxaliplatin as neoadjuvant therapy for patients with potentially curable metastatic colorectal cancer. *J Clin Oncol*. 2008;26(11):1830-1835.	314
69	Chun YS, et al. Association of computed tomography morphologic criteria with pathologic response and survival in patients treated with bevacizumab for colorectal liver metastases. *JAMA*. 2009;302(21):2338-2344.	314
70	Abdalla EK, et al. Extended hepatectomy in patients with hepatobiliary malignancies with and without preoperative portal vein embolization. *Arch Surg*. 2002;137(6):675-681.	313

71	Aloia TA, et al. Solitary colorectal liver metastasis: resection determines outcome. *Arch Surg*. 2006;141(5):460-467.	312
72	Van Hazel G, et al. Randomised phase 2 trial of SIR-Spheres plus fluorouracil/leucovorin chemotherapy versus fluorouracil/leucovorin chemotherapy alone in advanced colorectal cancer. *J Surg Oncol*. 2004;88(2):78-85.	303
73	Nakano H, et al. Sinusoidal injury increases morbidity after major hepatectomy in patients with colorectal liver metastases receiving preoperative chemotherapy. *Ann Surg*. 2008;247(1):118-124.	300
74	Jubb AM, et al. Impact of vascular endothelial growth factor-A expression, thrombospondin-2 expression, and microvessel density on the treatment effect of bevacizumab in metastatic colorectal cancer. *J Clin Oncol*. 2006;24(2):217-227.	298
75	Lee MT, et al. Phase I study of individualized stereotactic body radiotherapy of liver metastases. *J Clin Oncol*. 2009;27(10):1585-1591.	296
76	Kemeny MM, et al. Combined-modality treatment for resectable metastatic colorectal carcinoma to the liver: surgical resection of hepatic metastases in combination with continuous infusion of chemotherapy–an intergroup study. *J Clin Oncol*. 2002;20(6):1499-1505.	294
77	Iwatsuki S, et al. Hepatic resection for metastatic colorectal adenocarcinoma: a proposal of a prognostic scoring system. *J Am Coll Surg*. 1999;189(3):291-299.	292
78	Halazun KJ, et al. Elevated preoperative neutrophil to lymphocyte ratio predicts survival following hepatic resection for colorectal liver metastases. *Eur J Surg Oncol*. 2008;34(1):55-60.	292
79	Martin R, et al. Simultaneous liver and colorectal resections are safe for synchronous colorectal liver metastasis. *J Am Coll Surg*. 2003;197(2):233-242.	291
80	Steele G Jr, et al. A prospective evaluation of hepatic resection for colorectal carcinoma metastases to the liver: Gastrointestinal Tumor Study Group Protocol 6584. *J Clin Oncol*. 1991;9(7):1105-1112.	288
81	Adam R, et al. Repeat hepatectomy for colorectal liver metastases. *Ann Surg*. 1997;225(1):51-62.	284
82	Vogl TJ, et al. Malignant liver tumors treated with MR imaging-guided laser-induced thermotherapy: technique and prospective results. *Radiology*. 1995;196(1):257-265.	280

83	Hoyer M, et al. Phase II study on stereotactic body radiotherapy of colorectal metastases. *Acta Oncol*. 2006;45(7):823-830.	278
84	Rubbia-Brandt L, et al. Importance of histological tumor response assessment in predicting the outcome in patients with colorectal liver metastases treated with neo-adjuvant chemotherapy followed by liver surgery. *Ann Oncol*. 2007;18(2):299-304.	275
85	Lorenz M, et al. Randomized trial of surgery versus surgery followed by adjuvant hepatic arterial infusion with 5-fluorouracil and folinic acid for liver metastases of colorectal cancer. German Cooperative on Liver Metastases (Arbeitsgruppe Lebermetastasen). *Ann Surg*. 1998;228(6):756-762.	274
86	Van Cutsem E, et al. Oral capecitabine vs intravenous 5-fluorouracil and leucovorin: integrated efficacy data and novel analyses from two large, randomised, phase III trials. *Br J Cancer*. 2004;90(6):1190-1197.	274
87	Jaeck D, et al. Long-term survival following resection of colorectal hepatic metastases. Association Française de Chirurgie. *Br J Surg*. 1997;84(7):977-980.	273
88	Tabernero J, et al. Phase II trial of cetuximab in combination with fluorouracil, leucovorin, and oxaliplatin in the first-line treatment of metastatic colorectal cancer. *J Clin Oncol*. 2007;25(33):5225-5232.	272
89	Jamison RL, et al. Hepatic resection for metastatic colorectal cancer results in cure for some patients. *Arch Surg*. 1997;132(5):505-511.	270
90	Wilson SM, et al. Surgical treatment of hepatic metastases from colorectal cancers. *Arch Surg*. 1976;111(4):330-334.	269
91	House MG, et al. Survival after hepatic resection for metastatic colorectal cancer: trends in outcomes for 1,600 patients during two decades at a single institution. *J Am Coll Surg*. 2010;210(5):744-755.	264
92	de Haas RJ, et al. R1 resection by necessity for colorectal liver metastases: is it still a contraindication to surgery?. *Ann Surg*. 2008;248(4):626-637.	262
93	Ince WL, et al. Association of k-ras, b-raf, and p53 status with the treatment effect of bevacizumab. *J Natl Cancer Inst*. 2005;97(13):981-989.	260
94	Kennedy AS, et al. Resin 90Y-microsphere brachytherapy for unresectable colorectal liver metastases: modern USA experience. *Int J Radiat Oncol Biol Phys*. 2006;65(2):412-425.	259

95	Amin Z, et al. Hepatic metastases: interstitial laser photocoagulation with real-time US monitoring and dynamic CT evaluation of treatment. *Radiology*. 1993;187(2):339-347.	257
96	Reddy SK, et al. Simultaneous resections of colorectal cancer and synchronous liver metastases: a multi-institutional analysis. *Ann Surg Oncol*. 2007;14(12):3481-3491.	256
97	Kemeny NE, et al. Hepatic arterial infusion versus systemic therapy for hepatic metastases from colorectal cancer: a randomized trial of efficacy, quality of life, and molecular markers (CALGB 9481). *J Clin Oncol*. 2006;24(9):1395-1403.	252
98	Leporrier J, et al. A population-based study of the incidence, management and prognosis of hepatic metastases from colorectal cancer. *Br J Surg*. 2006;93(4):465-474.	243
99	Kokudo N, et al. Proliferative activity of intrahepatic colorectal metastases after preoperative hemihepatic portal vein embolization. *Hepatology*. 2001;34(2):267-272.	241
100	Allen PJ, et al. Importance of response to neoadjuvant chemotherapy in patients undergoing resection of synchronous colorectal liver metastases. *J Gastrointest Surg*. 2003;7(1):109-117.	239
